# Comparative Psychological Evaluation of Individuals With and Without Cleft Lip and Palate: A Systematic Review and Meta-Analysis

**DOI:** 10.1192/bjo.2024.170

**Published:** 2024-08-01

**Authors:** Riham Eltayib, Danya Ibrahim

**Affiliations:** 1 University of Dundee, School of Dentistry, Dundee, United Kingdom; 2 Khartoum University, Faculty of Medicine, Khartoum, Sudan

## Abstract

**Aims:**

This study sought to assess and compare the psychological well-being of both children and adults with cleft lip and palate (CLP) in contrast to those without CLP. The focus was on self-satisfaction regarding facial appearance, popularity among peers, and self-esteem.

**Methods:**

This study was registered at the University of Dundee, including various study designs such as randomized controlled clinical trials, longitudinal, cross-sectional, and observational studies. Only studies concentrating on non-syndromic CLP in children and adults were considered. No limitations were set on age or language. Self-reports, including validated and unvalidated questionnaires, interviews, and observational/clinical assessments, were deemed suitable. Database searches were performed in Medline, Pubmed, Scopus, Cochrane, and Web of Science (January 2019). An electronic search yielded 334 results, with 74 articles meeting the inclusion criteria. After screening and risk of bias assessment, four articles were included in the qualitative analysis, three of which were included in the meta-analysis.

**Results:**

The review encompassed four studies conducted in China, Japan, Sweden, and the United States, involving 442 participants (non-CLP control group n = 305, CLP group n = 137). While the majority of individuals with CLP did not exhibit significant psychological issues, certain challenges were noted, particularly concerning speech or hearing difficulties, depression, anxiety, and interpersonal relationships. Age did not seem to correlate with the occurrence or severity of psychological problems in CLP patients, with gender playing a significant role, as females tended to be more sensitive to facial appearance. The level of self-satisfaction was not statistically significant between the two groups (OR = 0.85) while the non-CLP group was more likely to rate themselves as being more popular among their peers (OR = 1.48). Also, the non-CLP group has higher self-esteem than CLP patients (OR = 1.05).

**Conclusion:**

Limited evidence suggests that some individuals with cleft lip and palate may face psychological challenges, indicating a need for more structured approaches to assess the psychological well-being of CLP patients.

